# Evaluation of the SpO_2_/FiO_2_ ratio as a predictor of intensive care unit transfers in respiratory ward patients for whom the rapid response system has been activated

**DOI:** 10.1371/journal.pone.0201632

**Published:** 2018-07-31

**Authors:** Won Gun Kwack, Dong Seon Lee, Hyunju Min, Yun Young Choi, Miae Yun, Youlim Kim, Sang Hoon Lee, Inae Song, Jong Sun Park, Young-Jae Cho, You Hwan Jo, Ho Il Yoon, Jae Ho Lee, Choon-Taek Lee, Yeon Joo Lee

**Affiliations:** 1 Division of Pulmonary and Critical Care Medicine, Department of Internal Medicine, Seoul National University Bundang Hospital, Seongnam, Republic of Korea; 2 Department of Internal Medicine, Seoul National University College of Medicine, Seoul, Republic of Korea; 3 Interdepartment of Critical Care Medicine, Seoul National University Bundang Hospital, Seongnam, Republic of Korea; 4 Department of Anesthesiology, Seoul National University Bundang Hospital, Seongnam, Republic of Korea; 5 Department of Emergency Medicine, Seoul National University Bundang Hospital, Seongnam, Republic of Korea; National Yang-Ming University, TAIWAN

## Abstract

Efforts to detect patient deterioration early have led to the development of early warning score (EWS) models. However, these models are disease-nonspecific and have shown variable accuracy in predicting unexpected critical events. Here, we propose a simpler and more accurate method for predicting risk in respiratory ward patients. This retrospective study analyzed adult patients who were admitted to the respiratory ward and detected using the rapid response system (RRS). Study outcomes included transfer to the intensive care unit (ICU) within 24 hours after RRS activation and in-hospital mortality. Prediction power of existing EWS models including Modified EWS (MEWS), National EWS (NEWS), and VitalPAC EWS (ViEWS) and SpO_2_/FiO_2_ (SF) ratio were compared to each other using the area under the receiver operating characteristic curve (AUROC). Overall, 456 patients were included; median age was 75 years (interquartile range: 65–80) and 344 (75.4%) were male. Seventy-three (16.0%) and 79 (17.3%) patients were transferred to the ICU and died. The SF ratio displayed better or comparable predictive accuracy for unexpected ICU transfer (AUROC: 0.744) compared to MEWS (0.744 vs. 0.653, *P* = 0.03), NEWS (0.744 vs. 0.667, *P* = 0.04), and ViEWS (0.744 vs. 0.675, *P* = 0.06). For in-hospital mortality, although there was no statistical difference, the AUROC of the SF ratio (0.660) was higher than that of each of the preexisting EWS models. In comparison with the preexisting EWS models, the SF ratio showed better or comparable predictive accuracy for unexpected ICU transfers in the respiratory wards.

## Introduction

Respiratory diseases are among the leading causes of the disability- adjusted life-years lost and mortality worldwide, accounting for one-quarter of the total global mortality [1]. In Korea, pneumonia has been the most common cause of hospital admission over the of last six years [2]. Patients admitted respiratory ward are more likely to need the assistance of a rapid response system (RRS) due to their vulnerable health status, including frequent desaturation, which can become critical. For these reasons, it is very important to predict acute deterioration of inpatients and initiate appropriate management in the respiratory ward.

Since the conception of the original early warning score (EWS) in the 1990s, various revised EWS such as Modified EWS (MEWS), National EWS (NEWS), and VitalPAC EWS (ViEWS) have been developed and used to discriminate the risk of catastrophic deterioration in general ward [3–6]. Although a number of studies reported that EWS systems can predict clinical outcomes, including unexpected intensive care unit (ICU) admission and cardiopulmonary arrest [7], there are remaining concerns that most EWS systems are disease-nonspecific and have relatively low predictive accuracy. Accordingly, new models with additional factors such as lactic acid or d-dimer levels, which have not been included in traditional EWS models, have been developed and validated to improve accuracy according to targeted patient groups [8, 9]. There is also a need for tailored EWS models to ensure practical usefulness in the respiratory wards [10].

The noninvasive and continuous pulse oximetry saturation (SpO_2_) is frequently checked in general wards and is included in the standard monitoring required in most ICUs [11]. In a previous study, the SpO_2_/fraction of inspired oxygen (FiO_2_) ratio (or SF ratio) was correlated with partial pressure of arterial oxygen (PaO_2_)/ FiO_2_ ratio (or PF ratio), which is a severity index in acute lung injury and acute respiratory distress syndrome (ARDS) [12]. Additionally, the SF ratio based ARDS patients demonstrated similar clinical characteristics and outcomes, duration of ventilator support and ICU stay, when compared with the PF ratio based ARDS patients [13]. In light of these findings, the SF ratio may be considered an easily applicable tool for the early recognition of patients with the potential for acute deterioration. However, to our knowledge, there is no report that the discrimination power of the SF ratio alone, and in comparison with other models, is a predictor for patients admitted in the respiratory ward.

The present study aimed to evaluate the ability of the SF ratio to predict acute deterioration in patients in the respiratory ward and to compare its predictive power with conventional EWS models (MEWS, NEWS, and ViEWS).

## Materials and methods

### Study design and patients

We conducted a retrospective cohort study using the RRS registry of the Seoul National University Bundang Hospital (SNUBH), a 1,360-bed tertiary care academic hospital, from January 2015 to August 2017. We extracted the data of patients admitted in the respiratory ward from the RRS registry. Detailed demographics, clinical data, and physical findings at RRS activation were ascertained through an electronic medical record (EMR) chart review. Collected variables included age, sex, Charlson comorbidity index score [14], cause of admission, systolic and diastolic blood pressure, heart rate, respiratory rate, body temperature, conscious level (AVPU: alert, responds to voice, responds to pain, and unresponsive), SpO_2_, the presence of oxygen therapy, FiO_2_, patients disposition after the RRS intervention, length of hospital stay, and survival status at discharge. Subsequently, the SF ratio, MEWS, NEWS, and ViEWS were obtained. The MEWS, NEWS, and ViEWS were calculated by summing the total score according to each of the predefined categories (Table 1 and S1 and S2 Tables). The primary outcomes were unplanned ICU transfer within 24 hours after RRS activation and in-hospital mortality. We compared the various scores with the SF ratio using a receiver operating characteristic (ROC) curve analysis.

**Table 1:** Modified early warning score (MEWS).

Variables	MEWS						
	3	2	1	0	1	2	3
SBP (mmHg)	≤ 70	71–80	81–100	101–199		≥ 200	
Heart rate (bpm)		≤ 40	41–50	51–100	101–110	111–129	≥ 130
Respiratory rate (bpm)	< 9		9–14	15–20	21–29		≥ 30
Temperature (°C)	< 35			35–38.4		38.5	
AVPU Score				A	V	P	U

SBP, systolic blood pressure; A, alert; V, responds to verbal stimuli; P, responds to pain only; U, unresponsive to stimuli.

### Rapid response system design

Our hospital has operated a part-timed RRS since October 2012. The RRS team has a role to timely detect and manage deteriorating patients in general ward [15]. RRS activation is possible in two ways except when a cardiopulmonary resuscitation request (CPR) is announced. The main way in which RRS is activated is through an automatic EMR screening system based on 10 screening criteria, each with a predefined threshold, including physiological and laboratory parameters (S3 Table). When a patient meets any of the screening criteria s/he is presented to a monitoring board and dedicated RRS nurses conduct a primary check up on the patient. Patients are also identified via direct requests from healthcare providers including residents or nurses. In the present study, if one patient had multiple RRS activations over the one admission period, only the first event was included. In case of multiple admissions during the study period, the same inclusion/exclusion criteria were applied individually to each admission. Patient events were excluded if they were documented as a do not resuscitate (DNR) prior to RRS activation or if they had RRS activation for CPR.

### Ethical approval

The present study was performed in accordance with the Declaration of Helsinki. The Institutional Review Board (IRB) of the Seoul National University Bundang Hospital (SNUBH) approved the study (approved IRB No.: B-1801-447-105) and informed consent was waived due to the retrospective study design.

### Statistical analysis

Continuous data and categorical data were presented as medians and interquartile ranges (IQR) or mean ± standard deviation. All continuous and categorical variables were compared using a Student’s t-test or Mann–Whitney U test, and Chi-square test, respectively. The accuracy of each parameter or models for predicting ICU transfers and in-hospital mortality were quantified using the area under the ROC curve (AUROC) and comparisons for each AUROC were determined using the De Long’s test [16]. The Youden index point was calculated as the optimal threshold for the ROC analysis [17]. To assess reasonable cutoff values, sensitivity, specificity, positive predictive values (PPV), negative predictive values (NPV), positive likelihood ratio (PLR), and negative likelihood ratio (NLR) were also investigated. All data were statistically analyzed using SPSS 23.0 for Windows (SPSS, Chicago, IL, USA) and MedCalc 17.9.7 for Windows (MedCalc, Ostend, Belgium) and *P*-values of less than 0.05 were considered as significant.

## Results

During study period, 1018 patients required practical RRS, however 562 were ineligible based on the exclusion criteria (duplicated cases: 336; prior DNR: 153; CPR: 5; monitoring in transfer and simple procedure assistant: 68). Consequently, 456 patients were included in this study. The median age of enrolled participants was 75 (IQR: 65–80) years and 344 (75.4%) were male. The median SF ratio was 294 (IQR: 209–388). ICU transfer within the 24 hours following RRS activation and in-hospital mortality occurred in 90 (19.7%) and 79 (17.3%) patients, respectively (Table 2).

**Table 2:** Baseline characteristics and clinical outcomes of RRS-activated patients in respiratory wards.

Variables	
Age, year	75 (65–80)
Sex, male, n (%)	344 (75.4)
Charlson comorbidity index	5 (4–6)
Systolic blood pressure, mmHg	117 (103–135)
Diastolic blood pressure, mmHg	66 (57–74)
Heart rate, beats/min	98 (82–110)
Respiratory rate, breath/min	24 (20–26)
Temperature, °C	36.8 (36.5–37.3)
AVPU score*	0 (0–1)
SF ratio^†^	294 (209–388)
MEWS, score range (0 ~ 14)	3 (2–4)
NEWS, score range (0 ~ 20)	8 (5–10)
ViEWS, score range (0 ~ 21)	9 (6–10)
ICU transfer (within 24 hr), n (%)	90 (19.7)
In-hospital mortality, n (%)	79 (17.3)

Data are presented as medians (interquartile range) or number (percentage) of patients. MEWS, Modified Early Warning Score; NEWS, National Early Warning Score; ViEWS, VitalPAC Early Warning Score; ICU, intensive care unit.

*AVPU: alert, responds to voice, responds to pain, and unresponsive

†SF ratio: SpO_2_/FiO_2_ ratio.

The differences in characteristics and calculated predicting parameters at the time of RRS activation based on the patient’ disposition after RRS intervention and in-hospital mortality were identified (S4 and S5 Tables). Compared to the general ward group, the ICU transfer group had a lower SF ratio (median 165 vs. 320, *P* < 0.01) and a higher score for all of the conventional EWS models including MEWS, NEWS, and ViEWS (*P* < 0.01). The mortality group also had a lower SF ratio (median 217 vs. 307, *P* < 0.01) and a higher score for all of conventional EWS models than those who survived (*P* < 0.01). Additionally, the Charlson comorbidity index, chronic respiratory disease, presence of a neoplasm, and interstitial lung disease (cause of admission) were also significantly higher in the mortality group (*P* < 0.01). Multivariate analysis, adjusted for age, sex, Charlson comorbidity index, chronic respiratory disease, presence of a neoplasm, and interstitial lung disease (cause of admission) for each predictive indicator, showed that the SF ratio, MEWS, NEWS and ViEWS had significant association with ICU transfers and in-hospital mortality (Table 3).

**Table 3:** Predictive values for intensive care unit transfers and mortality.

Variables	ICU transfer (n = 90)				Mortality (n = 79)		
	OR	95% CI		*P* value	OR	95% CI	*P* value
MEWS	1.424	1.248–1.625	< 0.01		1.369	1.188–1.578	< 0.01
NEWS	1.254	1.150–1.368	< 0.01		1.169	1.068–1.280	< 0.01
ViEWS	1.256	1.154–1.367	< 0.01		1.163	1.065–1.269	< 0.01
SF ratio*	0.992	0.989–0.994	< 0.01		0.996	0.993–0.998	< 0.01

Adjusted with age, sex, Charlson comorbidity index, chronic respiratory disease, neoplasm, interstitial lung disease (cause of admission). MEWS, Modified Early Warning Score; NEWS, National Early Warning Score; ViEWS, VitalPAC Early Warning Score; ICU, intensive care unit.

*SF ratio: SpO_2_/FiO_2_ ratio.

**Fig 1:**
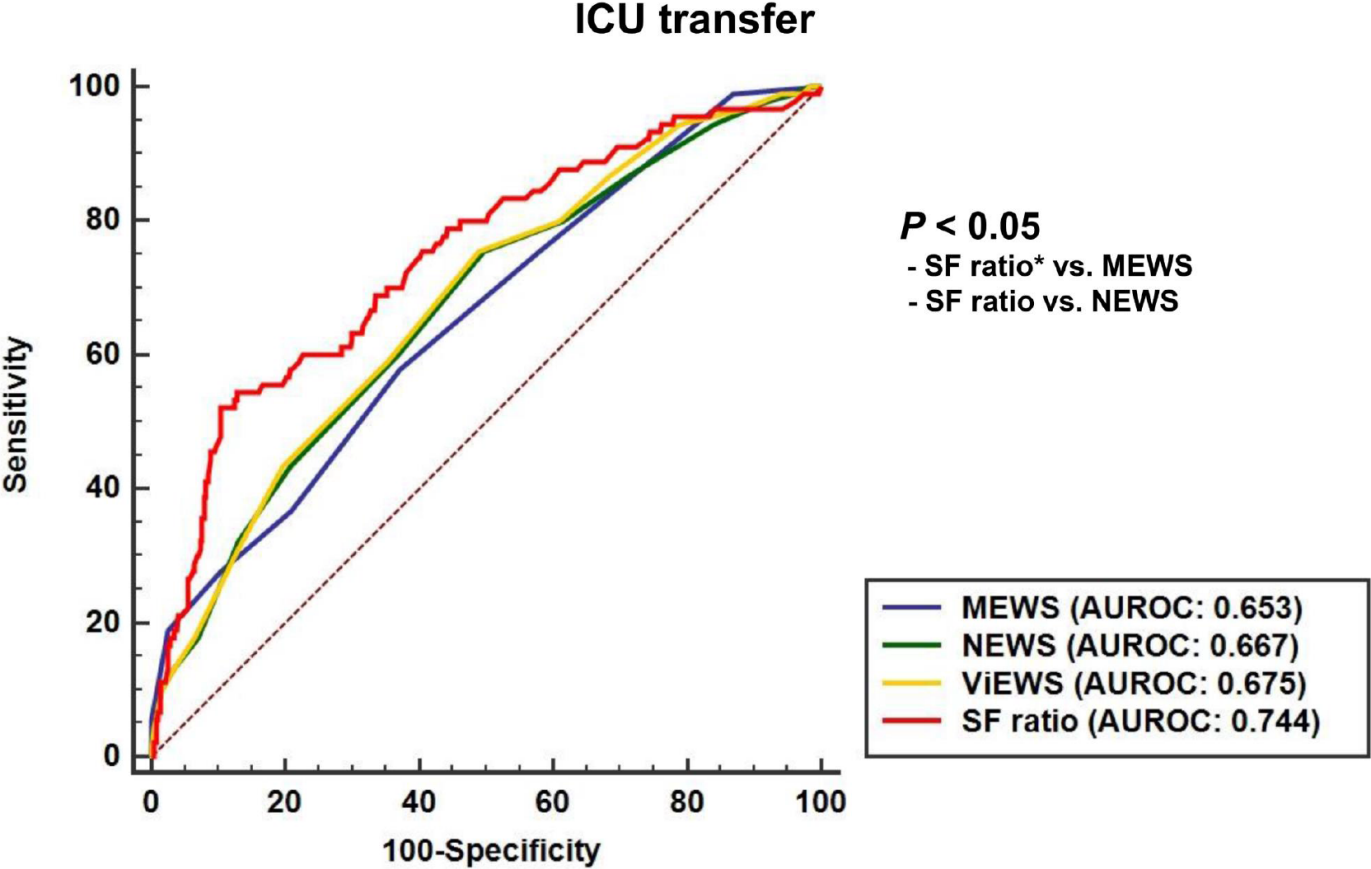
Comparison of the area of under the receiver operating characteristics curve for intensive care unit transfers within 24 hours of rapid response system activation. MEWS, Modified Early Warning Score; NEWS, National Early Warning Score; ViEWS, VitalPAC Early Warning Score; AUROC, area under the receiver operating characteristic curve; ICU, intensive care unit. *SF ratio: SpO_2_/FiO_2_ ratio.

**Fig 2:**
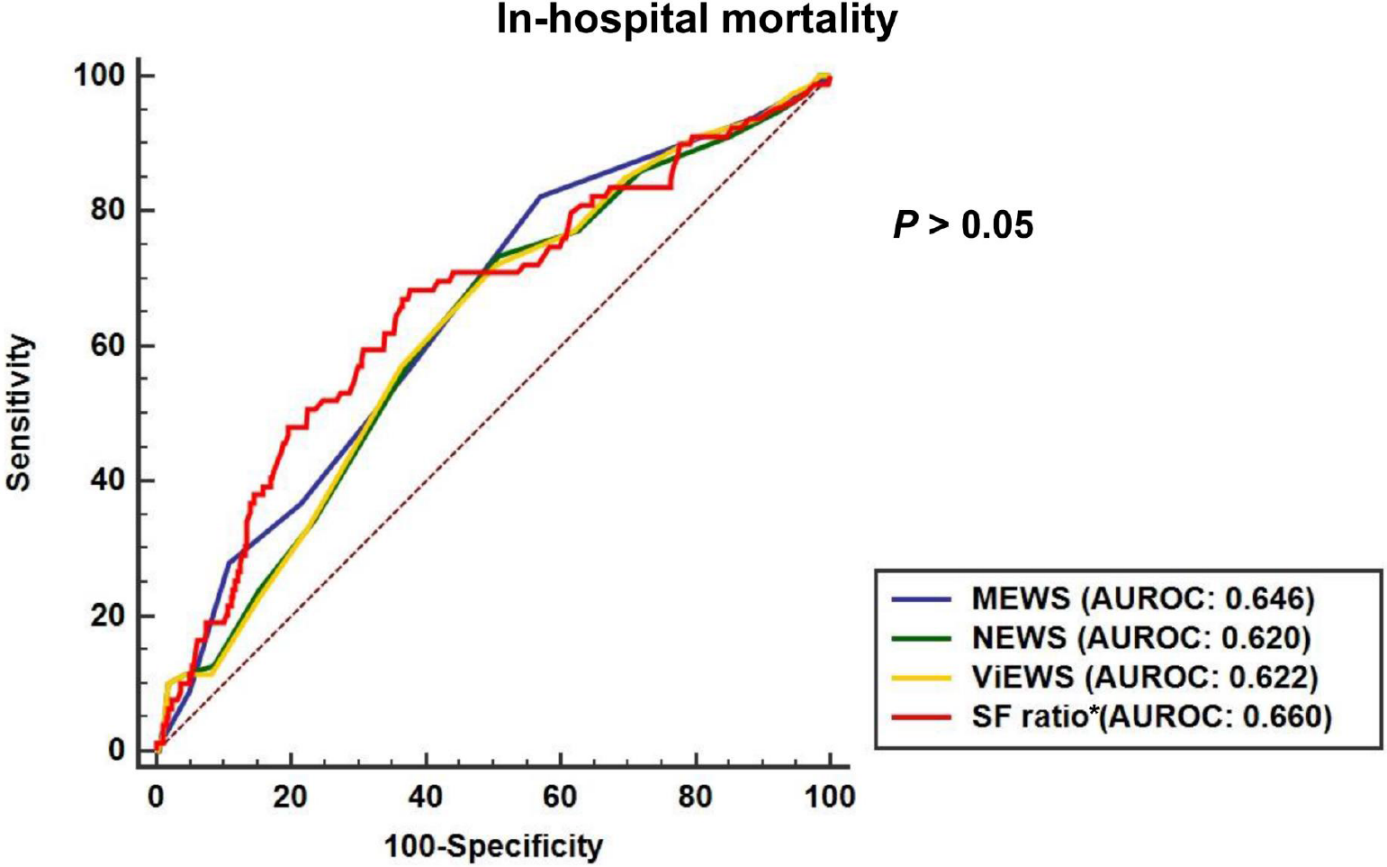
Comparison of the area of under the receiver operating characteristics curve for in-hospital mortality. MEWS, Modified Early Warning Score; NEWS, National Early Warning Score; ViEWS, VitalPAC Early Warning Score; AUROC, area under the receiver operating characteristic curve. *SF ratio: SpO_2_/FiO_2_ ratio.

In the ROC curve analysis (Fig 1) for ICU transfers, the AUROC of the SF ratio (0.744; 95% confidence interval [CI] 0.684 to 0.803) was significantly higher than that of MEWS (0.653; 0.59 to 0.716), and NEWS (0.667; 0.605 to 0.73). The SF ratio also had a higher AUROC compared to ViEWS, however this difference was not significant (0.744 vs. 0.675, *P* = 0.06). For in-hospital mortality (Fig 2), although there were no significant differences (*P* > 0.05), the AUROC of the SF ratio (0.660; 0.614 to 0.703) was higher than that any of each EWS models.

For ICU transfers, diagnostic performance of the SF ratio including sensitivity, specificity, PPV, NPV, PLR, and NLR were assessed (Table 4). The Youden index cutoff of 170 had a sensitivity, specificity, PPV, and NPV of 52.2%, 89.6%, 55.3%, and 88.4%, respectively. On the contrary, a cutoff of 300 showed the highest sensitivity, with a specificity of more than 50%. Using 300 as the cutoff resulted in a sensitivity, specificity, PPV, and NPV of 78.8%, 53.8%, 29.8%, and 91.3%, respectively.

**Table 4:** Predictive values for intensive care unit transfers.

Variables	Sensitivity	Specificity	PPV	NPV	PLR	NLR
SF ratio* ≤ 170^†^	52.2 (41.4–62.9)	89.6 (86.0–82.5)	55.3 (44.1–66.1)	88.4 (84.7–91.5)	5.0 (3.5–7.2)	0.53 (0.3–0.7)
SF ratio ≤ 200	54.4 (43.6–65.0)	83.8 (79.1–87.0)	46.7 (36.9–56.7)	88.3 (84.5–91.5)	3.38 (2.5–4.6)	0.54 (0.4–0.7)
SF ratio ≤ 250	65.6 (54.8–75.3)	67.7 (62.7–72.5)	33.3 (26.4–40.8)	88.9 (84.6–92.3)	2.03 (1.6–2.5)	0.51 (0.4–0.7)
SF ratio ≤ 300	78.8 (69.0–86.8)	53.8 (48.6–59.0)	29.8 (24.1–36.1)	91.3 (86.7–94.7)	1.71 (1.5–2.0)	0.39(0.3–0.6)

Sensitivity, specificity, PPV, and NPV were presented percentage (95% CIs). PLR and NLR were presented ratio (95% CIs). CI, confidence interval; PPV, positive predictive value; NPV, negative predictive value; PLR, positive likelihood ratio; NLR, negative likelihood ratio.

*SF ratio: SpO_2_/FiO_2_ ratio

†Youden index point.

## Discussion

The present study indicated that, as compared with several preexisting EWS systems (MEWS, NEWS and ViEWS), the SF ratio offered superior or equal prediction accuracy for ICU transfers from the respiratory ward. Additionally, the SF ratio had greater prediction accuracy for in-hospital mortality than each of the conventional EWS models, however this difference was not statistically significant. To the best of our knowledge, this is the first study to evaluate the SF ratio as a predictor of unplanned ICU transfers and in-hospital mortality in patients admitted in a respiratory ward.

Early recognition of a critical deterioration can be a matter of life or death in the practical clinical field. The decision regarding whether a patient needs intensive care is important and always difficult. Furthermore, if the available ICU resources are limited, the decision becomes even more difficult. Therefore, useful tools as predictors of acute deterioration have been required to assist healthcare providers with precise decision making. Previous studies have found that most deteriorating patients already showed changes in their vital signs before the 6- to 24-hour period that preceded the catastrophic developments in their cases, such as unexpected ICU transfer or cardiopulmonary arrest [18–20]. For this reason, various EWS models based on physiologic parameters have been suggested and applied. However, in order for these instruments to be actually used in clinical practice, prediction accuracy and simplicity should be taken into account. For improvement in predicting accuracy, diverse modifications such as changes in weighting scores and additional factors have been piloted based on specific diseases or the locations that patients presented. Yoo *et al*. reported the combination of MEWS and blood lactate was more accurate than MEWS alone in prediction of ICU transfers in patients with severe sepsis/septic shock [21]. Jo *et al*. calculated score that was the sum of ViEWS and blood lactate, which are referred to as ViEW-L score. In patients admitted to the medical ICU via the emergency department, ViEW-L score offered the same or better predictive than ViEWS alone, simplified acute physiology scores (SAPS) II, and SAPS III for hospital mortality [22]. The combination of NEWS and D-dimer also appears to identify patients at risk of mortality within 30 days of admission [23]. Furthermore, using the sum of simplified regression weights, gastrointestinal early warning score (EWS-GI) composed of physiologic and laboratory parameters was suggested as a predictor for ICU transfers in gastroenterology wards [24].

Most of the previous modifications of EWS were more focused on the improvement of predictive accuracy than simplicity for easy application. In the present study, we found that the ability of the SF ratio for the prediction of ICU transfers and in-hospital mortality was better or comparable to conventional EWS models. Our findings highlight the importance of achieving a balance between improvements in accuracy and simplicity. The inclusion of more associated factors improved the accuracy in most, but not all cases. In the present study, the combination MEWS and the SF ratio using predicted probability exhibited more predictive accuracy than not only conventional EWS models but also the SF ratio for ICU transfers and in-hospital mortality (see online supplementary file S1 and S2 Figs). However, in actual clinical practice, score calculation according to complex scoring categories or formula is very difficult, even if an automatic calculation system can be employed to assist with the calculation. This might therefore pose as a barrier to wider application. The decision regarding the need for the introduction of further intensive care should takes into account the patient’s condition and clinical markers in a comprehensive manner. Risk factors and EWS models cannot be absolute criterion, but rather auxiliary tools. We do not anticipate that the SF ratio should be an absolute criterion, replacing the already existing EWS. Additionally, the SF ratio does not provide PaCO_2_ or acid-base status, two other potentially important clinical findings that reflect the patient’s state. Therefore, we suggest that the SF ratio could be a comparable or additional tool to be implemented for decision making in the respiratory ward. In the future, studies on new independent predictive factors or modified EWS should consider not only the improvement of accuracy but also user-friendly models as auxiliary tools. Furthermore, validation of the SF ratio for various situations including mixed or other patient populations, such as medical and surgical, is needed. Additionally, there is a need for well controlled studies regarding treatment options, such as the frequency of ICU treatment and definitive DNR state during hospitalization, in order to investigate more accurate predictive power for prognosis including in-hospital mortality. Such findings are expected to be useful and interesting.

We investigated the performance of SF ratio cutoff values of 170 (which was selected based on Youden’s index) and 300 for predicting unexpected ICU transfers. Using SF ratio categories of ≤170 vs. >170, we observed a higher specificity (89.6%) than sensitivity (52.2%), and the PLR and NLR values were 5.0 and 0.53, respectively. In contrast, using SF ratio categories of ≤300 vs. >300, we observed a higher sensitivity (78.8%) than specificity (53.8%), and the PLR and NLR values were 1.71 and 0.39, respectively. Based on these findings, a cutoff value of 170 could be used as a rule-in point and a cutoff value of 300 could be used as a rule-out point [25]. Furthermore, the previous study which evaluated the correlation between the SF ratio and the PF ratio, found that SF ratios of 235 and 315 corresponded to PF ratios of 200 and 300, respectively. These are the oxygenation criteria for defining moderate and mild ARDS according to the Berlin definition [26]. It is interesting that the suggested cutoff values in the present study were similar to the PF ratio criteria as the severity index of ARDS. By validating proper cutoff values, a reasonable tool for decision making could be devised. Still, it is necessary to determine whether it is more important to focus on rule-in or rule-out points as cutoff values for ICU transfers, depending on the facilities of individual ICUs.

Our study was subject to several limitations. First, there might be the difference between the real SF ratio and the estimated SF ratio. In present study, FiO_2_ was estimated based on the amount of O_2_ supply according to the type of tools and this could result in the difference between the real supplied oxygen FiO_2_ and estimated FiO_2_ with the exception of high flow nasal cannula. Furthermore, for an SpO_2_ value of over 97%, the oxyhemoglobin dissociation curve was nearly flat [27]. We cannot be sure that the oxygen supply was titrated appropriately in the patients with SpO_2_ values over 97%. Second, the RRS registry of our institution was constructed prospectively, and we analyzed data extracted from the registry. Therefore, the data were relatively accurate. However, there may be factors associated with ICU transfer and mortality that were not investigated due to the study’s retrospective nature. The presence subsequent RRS activations following the first activation and a patient’s history of receiving CPR are two such factors that may need to be evaluated in the future. Finally, we only compared the SF ratio with conventional EWS systems, which only consisted of physiologic variables. To evaluate the usefulness of the SF ratio, studies should also compare it with newly derived EWS models, such as recent combinations of preexisting EWS models with additional factors.

In conclusion, in the respiratory ward, compared to the preexisting EWS models including MEWS, NEWS and ViEWS, the SF ratio is simple formula which showed better or comparable predictive accuracy for unexpected ICU transfers. Therefore, the SF ratio with cutoff values of 170 and 300, can play a role in predicting acute deterioration and in communication along RRS chain. Future well controlled studies of the prognosis based on SF ratios are needed to investigate the actual clinical benefits.

## Supporting information

S1 Table National early warning score (NEWS). SBP, systolic blood pressure; A alert, V responds to verbal stimuli; P, responds to pain only; U, unresponsive to stimuli.
(DOCX)

S2 Table VitalPAC early warning score (ViEWS). SBP, systolic blood pressure; A, alert, V responds to verbal stimuli; P, responds to pain only; U, unresponsive to stimuli.
(DOCX)

S3 Table Rapid response system activation criteria. SBP, systolic blood pressure; HR, heart rate; RR, respiratory rate; BT, body temperature; SpO_2_, pulse oximetry saturation; pH, potential of hydrogen; PaCO_2_, partial pressure of carbon dioxide; PaO_2_, partial pressure of oxygen.
(DOCX)

S4 Table Comparisons based on patients disposition. Data are presented as medians (interquartile range) or numbers (percentage) of patients ICU, intensive care unit; CAD, coronary artery disease; CVA, cerebrovascular accident definition; COPD, chronic obstructive pulmonary disease; MEWS, Modified Early Warning Score; NEWS, National Early Warning Score; ViEWS, VitalPAC Early Warning Score; SD, standard deviation; CRP, C-reactive protein. *SF ratio: SpO_2_/FiO_2_ ratio.
(DOCX)

S5 Table Comparison based on survival. Data are presented as medians (interquartile range) or numbers (percentage) of patients. CAD, coronary artery disease; CVA, cerebrovascular accident definition; COPD, chronic obstructive pulmonary disease; MEWS, Modified Early Warning Score; NEWS, National Early Warning Score; ViEWS, VitalPAC Early Warning Score; SD, standard deviation; CRP, C-reactive protein. *SF ratio: SpO_2_/FiO_2_ ratio.
(DOCX)

S1 Fig Comparison of the area of under the receiver operating characteristics curve for intensive care unit transfers within 24 hours of rapid response system activation. MEWS, Modified Early Warning Score; NEWS, National Early Warning Score; ViEWS, VitalPAC Early Warning Score; AUROC, area under the receiver operating characteristic curve; ICU, intensive care unit. *MEWS-SF ratio: the combination MEWS and SF ratio was calculated using predicted probability; †SF ratio: SpO_2_/FiO_2_ ratio.
(DOCX)

S2 Fig Comparison of the area of under the receiver operating characteristics curve for in-hospital mortality. MEWS, Modified Early Warning Score; NEWS, National Early Warning Score; ViEWS, VitalPAC Early Warning Score; AUROC, area under the receiver operating characteristic curve. *MEWS-SF ratio: the combination MEWS and SF ratio was calculated using predicted probability; †SF ratio: SpO_2_/FiO_2_ ratio.
(DOCX)
