# Supplementary material for: Evaluation of the SpO2/FiO2 ratio as a predictor of intensive care unit transfers in respiratory ward patients for whom the rapid response system has been activated
Source: PLoS One. 2018 Jul 31;13(7):e0201632. doi: 10.1371/journal.pone.0201632 (PMC6067747; doi:10.1371/journal.pone.0201632)
Supplement: S1 Table — SBP, systolic blood pressure; A alert, V responds to verbal stimuli; P, responds to pain only; U, unresponsive to stimuli. (DOCX) [file pone.0201632.s001.docx]

Table 1. National early warning score (NEWS)

| Variables | NEWS | | | | | | |
| --- | --- | --- | --- | --- | --- | --- | --- |
|  | 3 | 2 | 1 | 0 | 1 | 2 | 3 |
| SBP (mmHg) | ≤90 | 91-100 | 101-110 | 111-219 |  |  | ≥220 |
| Heart rate (bpm) | ≤40 |  | 41-50 | 51-90 | 91-110 | 111-130 | ≥131 |
| Respiratory rate (bpm) | ≤8 |  | 9-11 | 12-20 |  | 21-24 | ≥25 |
| Temperature (°C) | ≤35 |  | 35.1-36.0 | 36.1-38.0 | 38.1-39.0 | ≥39.1 |  |
| AVPU Score |  |  |  | A |  |  | V, P, U |
| Added O_2_ |  | Yes |  | No |  |  |  |

SBP, systolic blood pressure; A, alert; V, responds to verbal stimuli; P, responds to pain only; U, unresponsive to stimuli.
