# Supplementary material for: Evaluation of the SpO2/FiO2 ratio as a predictor of intensive care unit transfers in respiratory ward patients for whom the rapid response system has been activated
Source: PLoS One. 2018 Jul 31;13(7):e0201632. doi: 10.1371/journal.pone.0201632 (PMC6067747; doi:10.1371/journal.pone.0201632)
Supplement: S3 Table — SBP, systolic blood pressure; HR, heart rate; RR, respiratory rate; BT, body temperature; SpO2, pulse oximetry saturation; pH, potential of hydrogen; PaCO2, partial pressure of carbon dioxide; PaO2, partial pressure of oxygen. (DOCX) [file pone.0201632.s003.docx]

Table 3. Rapid response system activation criteria

| Variable |
| --- |
| Criteria 1 SBP < 90 mmHg |
| Criteria 2 HR < 50, > 140 /min |
| Criteria 3 RR < 10, > 30 /min |
| Criteria 4 BT > 39, < 36℃ |
| Criteria 5 SpO_2_ < 90% |
| Criteria 6 pH < 7.25 |
| Criteria 7 PaCO_2_ > 50 mmHg |
| Criteria 8 PaO_2_ < 55 mmHg |
| Criteria 9 Lactic acid > 4 mmol/L |
| Criteria 10 Total CO_2_ < 15 mmol/L |

SBP, systolic blood pressure; HR, heart rate; RR, respiratory rate; BT, body temperature; SpO2, pulse oximetry saturation; pH, potential of hydrogen; PaCO2, partial pressure of carbon dioxide; PaO2, partial pressure of oxygen.
