# Supplementary material for: Evaluation of the SpO2/FiO2 ratio as a predictor of intensive care unit transfers in respiratory ward patients for whom the rapid response system has been activated
Source: PLoS One. 2018 Jul 31;13(7):e0201632. doi: 10.1371/journal.pone.0201632 (PMC6067747; doi:10.1371/journal.pone.0201632)
Supplement: S4 Table — Data are presented as medians (interquartile range) or numbers (percentage) of patients ICU, intensive care unit; CAD, coronary artery disease; CVA, cerebrovascular accident definition; COPD, chronic obstructive pulmonary disease; MEWS, Modified Early Warning Score; NEWS, National Early Warning Score; ViEWS, VitalPAC Early Warning Score; SD, standard deviation; CRP, C-reactive protein. *SF ratio: SpO2/FiO2 ratio. (DOCX) [file pone.0201632.s004.docx]

Table 4. Comparisons based on patient disposition

| Variables | General ward  (n = 366) | ICU transfer  (n = 90) | *P* value |
| --- | --- | --- | --- |
| Age, years, median (IQR) | 75 (65-80) | 75 (65-80) | 0.92 |
| Sex, male | 276 (75.4) | 68 (75.6) | 0.98 |
| Charlson comorbidity index | 5 (3-6) | 5 (4-6) | 0.56 |
| Hypertension | 100 (27.3) | 19 (21.1) | 0.23 |
| Diabetes mellitus | 101 (27.6) | 22 (24.4) | 0.55 |
| CAD | 15 (4.1) | 3 (3.3) | 0.74 |
| CVA | 44 (12.0) | 13 (14.4) | 0.53 |
| Chronic respiratory disease | 160 (43.7) | 39 (43.3) | 0.95 |
| Chronic renal disease | 39 (10.7) | 4 (4.4) | 0.07 |
| Chronic liver disease | 21 (5.7) | 6 (6.7) | 0.74 |
| Neoplasm | 87 (23.8) | 26 (28.9) | 0.31 |
| Cause of admission |  |  |  |
| Pneumonia | 185 (50.5) | 55 (61.1) | 0.07 |
| COPD | 40 (10.9) | 6 (6.7) | 0.23 |
| Interstitial lung disease | 33 (9.0) | 7 (7.8) | 0.71 |
| Lung cancer | 21 (5.7) | 4 (4.4) | 0.63 |
| Pulmonary tuberculosis | 15 (4.1) | 5 (5.6) | 0.55 |
| SF ratio* | 320 (225-400) | 165 (105-284) | < 0.01 |
| MEWS | 3 (2-4) | 4 (3-6) | < 0.01 |
| NEWS | 7 (5-9) | 9 (8-11) | < 0.01 |
| ViEWS | 8 (6-10) | 10 (9-12) | < 0.01 |
| Leucocytes/μL, mean ± SD (n = 334) | 8,727 ± 7,801  (n = 256) | 7,152 ± 8,260  (n = 78) | 0.14 |
| CRP, mg/L, mean ± SD  (n = 283) | 9.75 ± 8.11  (n = 213) | 11.26 ± 7.81  (n = 70) | 0.17 |

Data are presented as medians (interquartile range) or numbers (percentage) of patients. ICU, intensive care unit; CAD, coronary artery disease; CVA, cerebrovascular accident definition; COPD, chronic obstructive pulmonary disease; MEWS, Modified Early Warning Score; NEWS, National Early Warning Score; ViEWS, VitalPAC Early Warning Score; SD, standard deviation; CRP, C-reactive protein. *SF ratio: SpO2/FiO2 ratio.
