# Supplementary material for: Evaluation of the SpO2/FiO2 ratio as a predictor of intensive care unit transfers in respiratory ward patients for whom the rapid response system has been activated
Source: PLoS One. 2018 Jul 31;13(7):e0201632. doi: 10.1371/journal.pone.0201632 (PMC6067747; doi:10.1371/journal.pone.0201632)
Supplement: S5 Table — Data are presented as medians (interquartile range) or numbers (percentage) of patients. CAD, coronary artery disease; CVA, cerebrovascular accident definition; COPD, chronic obstructive pulmonary disease; MEWS, Modified Early Warning Score; NEWS, National Early Warning Score; ViEWS, VitalPAC Early Warning Score; SD, standard deviation; CRP, C-reactive protein. *SF ratio: SpO2/FiO2 ratio. (DOCX) [file pone.0201632.s005.docx]

Table 5. Comparison based on survival

| Variables | Survived  (n = 377) | Died  (n = 79) | *P* value |
| --- | --- | --- | --- |
| Age, years | 75 (64-80) | 75 (68-80) | 0.54 |
| Sex, male | 279 (74.0) | 65 (82.3) | 0.12 |
| Charlson comorbidity index | 5 (3-6) | 5 (4-6) | < 0.01 |
| Hypertension | 102 (27.1) | 17 (21.5) | 0.31 |
| Diabetes mellitus | 104 (27.6) | 19 (24.1) | 0.52 |
| CAD | 14 (3.7) | 4 (5.1) | 0.58 |
| CVA | 52 (13.8) | 5 (6.3) | 0.07 |
| Chronic respiratory disease | 154 (40.8) | 45 (57.0) | < 0.01 |
| Chronic renal disease | 37 (9.8) | 6 (7.6) | 0.54 |
| Chronic liver disease | 21 (5.6) | 6 (7.6) | 0.49 |
| Neoplasm | 80 (21.2) | 33 (41.8) | < 0.01 |
| Cause of admission |  |  |  |
| Pneumonia | 203 (53.8) | 37 (46.8) | 0.26 |
| COPD | 41 (10.9) | 5 (6.3) | 0.22 |
| Interstitial lung disease | 27 (7.2) | 13 (16.5) | < 0.01 |
| Lung cancer | 18 (4.8) | 7 (8.9) | 0.15 |
| Pulmonary tuberculosis | 15 (4.0) | 5 (6.3) | 0.35 |
| SF ratio* | 307 (220-392) | 217 (127-342) | < 0.01 |
| MEWS | 3 (2-4) | 4 (3-6) | < 0.01 |
| NEWS | 8 (5-9) | 9 (7-10) | < 0.01 |
| ViEWS | 9 (6-10) | 10 (8-11) | < 0.01 |
| Leucocytes/μL, mean ± SD (n = 334) | 8,733 ± 7,965  (n = 268) | 6,841 ± 7,636  (n = 66) | 0.08 |
| CRP, mg/L, mean ± SD  (n = 283) | 9.74 ± 8.11  (n = 229) | 11.77 ± 7.65  (n = 54) | 0.10 |

Data are presented as medians (interquartile range) or numbers (percentage) of patients. CAD, coronary artery disease; CVA, cerebrovascular accident definition; COPD, chronic obstructive pulmonary disease; MEWS, Modified Early Warning Score; NEWS, National Early Warning Score; ViEWS, VitalPAC Early Warning Score; SD, standard deviation; CRP, C-reactive protein. *SF ratio: SpO2/FiO2 ratio.
