# Supplementary material for: Evaluation of the SpO2/FiO2 ratio as a predictor of intensive care unit transfers in respiratory ward patients for whom the rapid response system has been activated
Source: PLoS One. 2018 Jul 31;13(7):e0201632. doi: 10.1371/journal.pone.0201632 (PMC6067747; doi:10.1371/journal.pone.0201632)
Supplement: S1 Fig — MEWS, Modified Early Warning Score; NEWS, National Early Warning Score; ViEWS, VitalPAC Early Warning Score; AUROC, area under the receiver operating characteristic curve; ICU, intensive care unit. *MEWS-SF ratio: the combination MEWS and SF ratio was calculated using predicted probability; †SF ratio: SpO2/FiO2 ratio. (DOCX) [file pone.0201632.s006.docx]

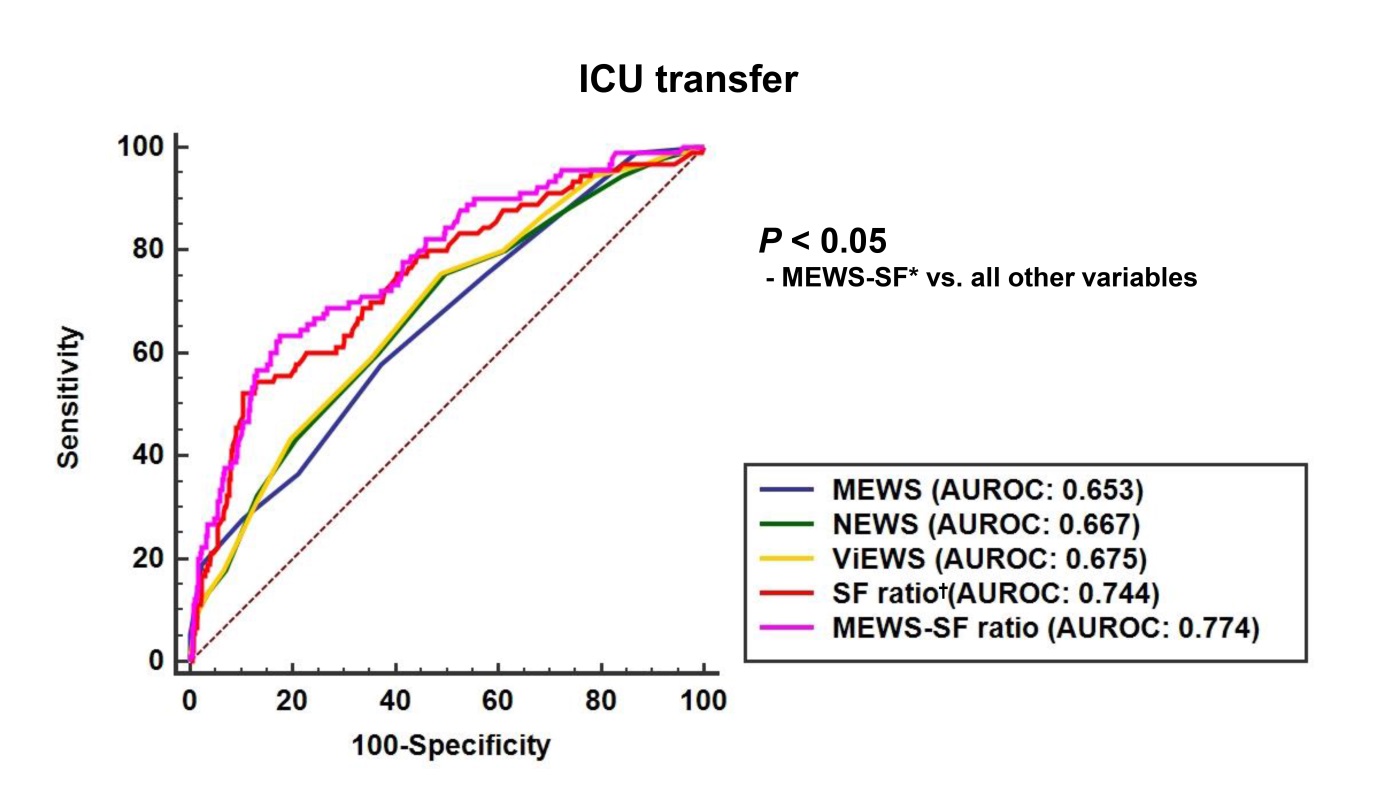


**Fig 1. Comparison of the area of under the receiver operating characteristics curve for intensive care unit transfers within 24 hours of rapid response system activation.** MEWS, Modified Early Warning Score; NEWS, National Early Warning Score; ViEWS, VitalPAC Early Warning Score; AUROC, area under the receiver operating characteristic curve; ICU, intensive care unit. ^*^MEWS-SF ratio: the combination MEWS and SF ratio was calculated using predicted probability; ^†^SF ratio: SpO_2_/FiO_2_ ratio.
