# Supplementary material for: Evaluation of the SpO2/FiO2 ratio as a predictor of intensive care unit transfers in respiratory ward patients for whom the rapid response system has been activated
Source: PLoS One. 2018 Jul 31;13(7):e0201632. doi: 10.1371/journal.pone.0201632 (PMC6067747; doi:10.1371/journal.pone.0201632)
Supplement: S2 Fig — MEWS, Modified Early Warning Score; NEWS, National Early Warning Score; ViEWS, VitalPAC Early Warning Score; AUROC, area under the receiver operating characteristic curve. *MEWS-SF ratio: the combination MEWS and SF ratio was calculated using predicted probability; †SF ratio: SpO2/FiO2 ratio. (DOCX) [file pone.0201632.s007.docx]

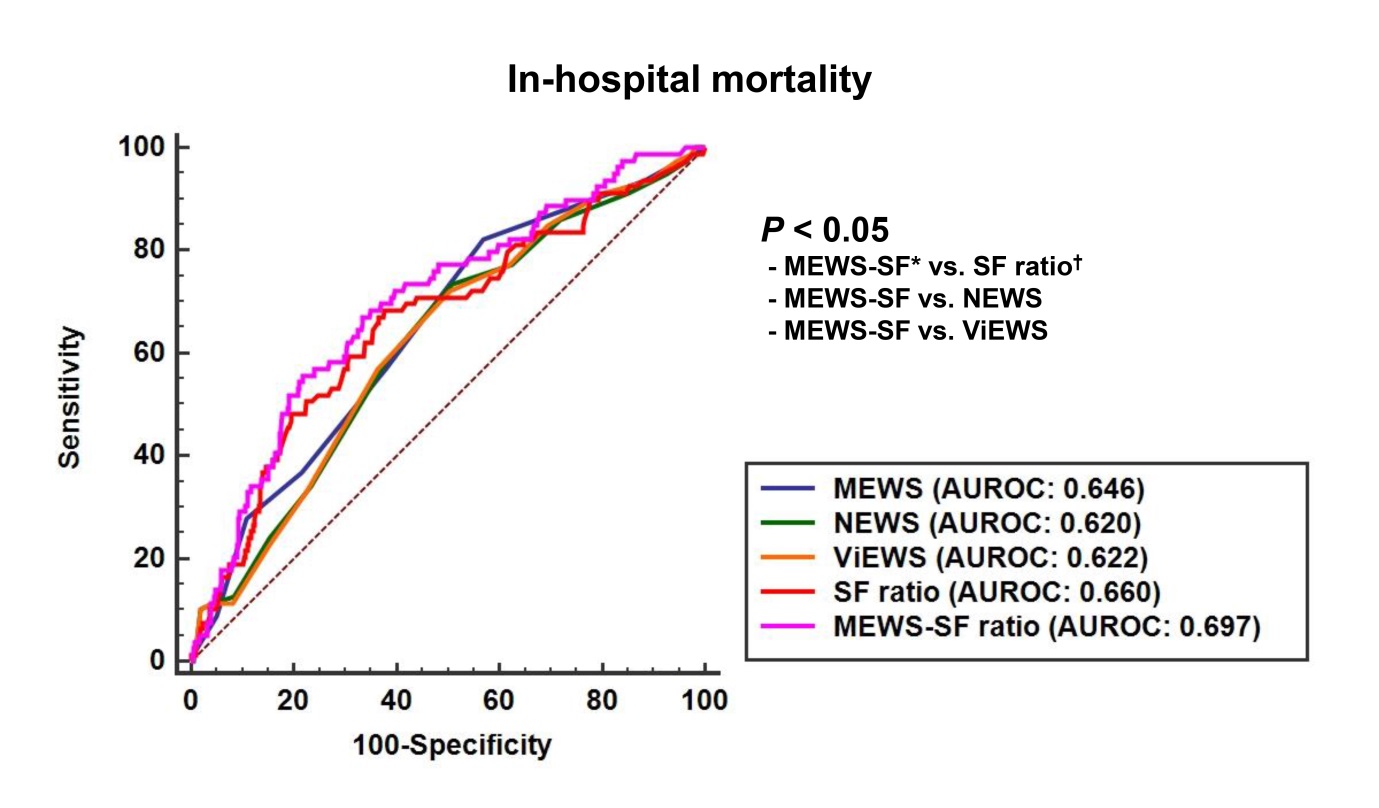


**Fig 2. Comparison of the area of under the receiver operating characteristics curve for in-hospital mortality.** MEWS, Modified Early Warning Score; NEWS, National Early Warning Score; ViEWS, VitalPAC Early Warning Score; AUROC, area under the receiver operating characteristic curve. *MEWS-SF ratio: the combination MEWS and SF ratio was calculated using predicted probability; †SF ratio: SpO2/FiO2 ratio.
